# Religious coping methods predict depression and quality of life among end-stage renal disease patients undergoing hemodialysis: a cross-sectional study

**DOI:** 10.1186/s12882-017-0619-1

**Published:** 2017-06-17

**Authors:** Paulo Roberto Santos, José Roberto Frota Gomes Capote Júnior, José Renan Miranda Cavalcante Filho, Ticianne Pinto Ferreira, José Nilson Gadelha dos Santos Filho, Stênio da Silva Oliveira

**Affiliations:** 1 Graduate Program in Health Sciences, Sobral Faculty of Medicine, Federal University of Ceará, Brazil, Rua Comandante Maurocélio Rocha Ponte 100, Sobral, CEP 62.042-280 Brazil; 2 Sobral Faculty of Medicine, Federal University of Ceará, Brazil, Rua Comandante Maurocélio Rocha Ponte 100, Sobral, CEP 62.042-280 Brazil; 3 Rua Tenente Amauri Pio, 380 apt. 900, Fortaleza, CE CEP 60.160-090 Brazil

**Keywords:** Spirituality, Religion and medicine, Renal dialysis, Depression, Quality of life

## Abstract

**Background:**

Poor quality of life (QOL) and a high prevalence of depression have been identified among end-stage renal disease (ESRD) patients undergoing hemodialysis (HD). We aimed to evaluate the associations between religious/spiritual (R/S) coping methods and both QOL and depression among ESRD patients undergoing hemodialysis (HD).

**Methods:**

The sample included 161 ESRD patients over 18 years of age who had been undergoing HD for more than 3 months. R/S coping methods were assessed using the Religious Coping Questionnaire (RCOPE). The RCOPE generates scores (from 1 to 5) for positive and negative R/S coping methods. The higher the score, the more frequent the use of that coping method. Depression was evaluated using the 20-item version of the Center for Epidemiologic Studies Depression Scale (CES-D). Scores on the CES-D range from 0 to 60. A cutoff of 18 was used to define depression. QOL was evaluated using the Medical Outcomes Study 36-Item Short Form Health Survey (SF-36); this survey was used to generate scores for the eight dimensions of QOL, which can vary from 0 (worst) to 100 (best).

**Results:**

We identified a depression prevalence of 27.3%. Positive R/S coping scores were higher among non-depressed than depressed patients (2.98 vs. 2.77; *p* = 0.037). Positive R/S coping scores were negatively correlated with depression scores (*r* = −0.200; *p* = 0.012) and were an independent protective factor for depression (OR = 0.13; CI 95% = 0.02-0.91; *p* = 0.039). Regarding QOL, a positive correlation was identified between positive R/S coping scores and scores related to general health (*r* = 0.171; *p* = 0.030) and vitality (*r* = 0.183; *p* = 0.019), and an inverse correlation was identified between negative R/S coping scores and scores in the social functioning (*r* = −0.191; *p* = 0.015) and mental health (*r* = −0.214; *p* = 0.006) dimensions. In addition, positive R/S coping scores were an independent predictor of higher scores in the bodily pain (β = 14.401; *p* = 0.048) and vitality (β = 12.580; *p* = 0.022) dimensions. In contrast, negative R/S coping scores independently predicted lower social functioning scores (β = −21.158; *p* = 0.017).

**Conclusions:**

Our results provide further evidence suggesting that R/S coping methods may be associated with QOL and depression among HD patients. In our opinion, the use of religious resources should be encouraged among HD patients, and psycho-spiritual interventions should be attempted to target religious struggles (negative R/S coping) in patients undergoing HD.

## Background

End-stage renal disease (ESRD) patients undergoing hemodialysis (HD) may experience the following stressors: time restrictions, dietary constraints, functional limitations, changes in sexual function, medication effects, awareness of impending death, and difficulties related to employment, social and family dynamics [1]. This set of problems may explain the poor quality of life (QOL) and high prevalence of depression identified among ESRD patients undergoing HD. In this regard, these patients have been reported to have poorer QOL than sufferers of other common chronic diseases, such as chronic heart failure, chronic lung disease, angina, arthritis, and even cancer [2]. Concerning depression, the existing literature suggests that the prevalence of depressive symptoms may reach 30% [3]. Moreover, QOL has not improved among HD patients over the last decade, and in the context of another medical illness, depression may be resistant to treatment [4, 5].

Against this background, it is crucial to ascertain potentially modifiable variables associated with QOL and depression. We have already identified some modifiable variables that could be the targets of interventions designed to improve QOL and decrease depressive symptoms, such as general coping style. We have also shown that problem-oriented coping was associated with better perception of QOL when compared with emotion-oriented coping [6]. Fortunately, general coping styles may be easily modified after few psychotherapy sessions [7]. Regarding depression, despite the difficulties of identifying the direction of causality between depression and sexuality, sexual dysfunction may serve as a condition that may be targeted among women using anti-depressive medications when these medications do not improve their depressive symptoms [8].

The associations between religiosity and spirituality and both better QOL and lower depression prevalence have been well recognized [9, 10]. Nevertheless, religious and spiritual beliefs have not been widely incorporated into clinical practice. For example, in countries where people tend to be very religious, such as Brazil, India and Indonesia, physicians are generally not trained in the use of religion or spirituality in daily practice [11]. Nonetheless, religious, and especially spiritual, coping methods may be modifiable either through spontaneous mechanisms or by intervention [12, 13]. If one type of religious/spiritual (R/S) coping method is more associated with better QOL and less depression than another coping method, it would be beneficial for HD patients to address R/S issues while receiving health care. Therefore, we aimed to evaluate the associations between R/S coping methods and both QOL and depression among ESRD patients undergoing HD.

## Methods

### Sample

The sample included ESRD patients undergoing HD in the only two dialysis centers in an area of 34,560 km^2^ (37.3 inhabitants/ km^2^) in the northern region of Ceará State, Northeast Brazil. Of the total of 188 ESRD patients undergoing HD during July 2015, we included 179 patients who were older than 18 years of age and had been undergoing maintenance HD for more than 3 months. Of these eligible patients, 161 were ultimately included. The reasons for exclusion among eligible patients were refusal to participate (8), cognitive impairment that precluded answering the questionnaire (6) and hospitalization (4). All patients were undergoing conventional HD (three four-hour sessions per week) with polysulfone dialyzers (maximum number of reuses = 12). Written informed consent was obtained from all participants, and the study was approved by the ethics committee of Vale Acaraú University, with which the dialysis centers are associated.

### Assessment of religious/spiritual (R/S) coping methods

We used the R/S coping scale entitled the Religious Coping Questionnaire (RCOPE), which was developed by Pargament, Koenig and Perez [14]. A version of the scale that was translated into Portuguese translated and culturally adapted to the Brazilian context was validated by Panzini and Bandeira [15]. In its Brazilian version, the instrument contains 87 items comprising positive (66 items) and negative (21 items) R/S coping methods. Positive R/S coping methods are those related to seeking spiritual help; offering help to each other; positively positioning oneself in front of God; and personal quest for spiritual knowledge. To be illustrative, examples of positive R/S coping scale items include the following: “working together with God as a partner to endure through problems” or “looking to God for strength, support, and guidance”. In contrast, negative R/S coping methods are related to negative evaluations of God; negatively positioning oneself in front of God; signs of spiritual tension; and conflict and struggles with God and others. Examples of negative R/S coping scale items include the following: “I feel like God has abandoned me”, or “I am angry with God for letting this happen to me”. The patients were asked to indicate how much or how frequently they used the aforementioned R/S methods of coping. Items were rated on a 5-point scale from 1 (not at all) to 5 (always). Both the positive and negative R/S coping scores were assessed as continuous variables. Higher positive R/S coping scores indicated the use of potentially helpful ways of coping, as they are scored based on questions comprising “positive” methods of R/S coping, while higher negative R/S coping scores can be seen as denoting harmful ways of coping, as they are scored based on items reflecting religious struggle. Overall scores were calculated as the mean of the positive and negative coping scores and varied from 1 to 5, with higher scores indicating more frequent use of each coping strategy.

### Depression

Depression was evaluated using the 20-item version of the Center for Epidemiologic Studies Depression Scale (CES-D) [16]; on this scale, the respondent reports the frequency of symptoms during the past week. The items on this scale address humor, psychosomatic symptoms and social interactions. Each of the 20 items on this instrument is assigned a value of 0, 1, 2 or 3, with higher scores indicating the presence and persistence of depressive symptoms. A score from 0 to 60 is calculated by summing the scores for each item. A score ≥ 16 has been used to define depression in the general population. We used a score ≥ 18 to define depression, as has been previously validated in a HD population [17].

### Quality of life

We used the validated Brazilian version of the Medical Outcomes Study 36-Item Short Form Health Survey (SF-36) to measure QOL [18]. This is a well-validated and widely used 36-item questionnaire that addresses eight dimensions of QOL. These dimensions include physical function, in which the patients’ performance in terms of daily activities is evaluated; role-physical, in which the impact of physical health on life is determined; bodily pain, in which pain level and its impact on normal daily activities is identified; general health, in which subjective perceptions of present and future health status and resistance to illness are evaluated; vitality, in which the patient’s feelings about his/her energy level, vitality, and moments of fatigue are determined; social functioning, in which the impact of health on routine social activities is assessed; role-emotional, in which the influence of emotional status on daily activities is identified; and mental health, in which humor and well-being, including depression and anxiety, are assessed. The SF-36 generates scores ranging from 0 (worst) to 100 (best) for each of the eight dimensions.

### Patient data

Demographic data, length of time on dialysis, religious affiliation, type of vascular access and underlying etiology of ESRD were obtained from the dialysis centers’ medical records. Underlying renal diseases were classified according to clinical criteria and not by histopathology. Patients were classified as “married” when they were in a stable union independent of civil status. Through a process of exclusion, all subjects who were not in a stable union were considered unmarried, including divorced/separated, widowed and single patients. Economic class was categorized according to criteria on the instrument issued by the Brazilian Association of Research Institutes [19]. This validated instrument has been used in marketing surveys and population censuses and grades economic class into five subgroups: A (best status) through E (worst status). In addition to income level, the criteria assessed for economic class include educational level of the head of household and ownership of household appliances. Each patient was assigned a low, medium or high risk index based on comorbidity, as described by Khan et al. [20]. Khan’s comorbidity index considers age and the presence of nine comorbidities: diabetes, myocardial infarction, angina pectoris, congestive heart failure, liver cirrhosis, obstructive pulmonary disease, systemic collagen disease, pulmonary fibrosis and visceral malignancy. Laboratory tests for serum creatinine, hemoglobin, albumin, calcium and phosphorus levels were performed. The dose of dialysis delivered was evaluated using a second-generation Kt/V equation, as described by Daugirdas [21].

### Statistical analyses

For the descriptive analyses, data are expressed as the mean ± standard deviation for continuous variables and absolute numbers and percentages for categorical variables. Comparisons of positive and negative R/S coping scores between depressed and non-depressed patients were performed using the Student’s t test. The Pearson’s correlation test was used to assess the correlation between R/S coping scores and scores generated using the SF-36 and CES-D. Multivariate linear regression analyses were performed to identify predictors of SF-36 and CES-D scores, and a multivariate logistic regression analysis was applied to identify predictors of the presence of depression. Positive and negative R/S coping scores and predictive variables traditionally used in HD samples (age, gender, time on dialysis, comorbidity) were included in the regression models. Although this was a cross-sectional study, we chose the term “predictor” because it has been widely used and is well understood. Statistical significance was considered at a *p*-value <0.05. All statistical analyses were performed using the SPSS version 22.0 program package.

## Results

The characteristics of the sample are depicted in Table 1. Overall, 117 (72.7%) non-depressed and 44 (27.3%) depressed patients were included in the study. Depressed patients had lower positive R/S coping scores (2.77 ± 0.57 vs. 2.98 ± 0.54; *p* = 0.037) and higher negative R/S coping scores (2.00 ± 0.47 vs. 1.70 ± 0.38; *p* < 0.001) than non-depressed patients. Positive R/S coping scores were negatively correlated with depression scores (*r* = −0.200, *p* = 0.012), while negative R/S coping scores were positively correlated with depression scores (*r* = 0.299, *p* < 0.001). Positive R/S coping score was an independent predictor of both depression score (β = −6.635; *p* = 0.004) and presence of depression (OR = 0.13; *p* = 0.039) (Tables 2 and 3). Additionally, negative R/S coping score was an independent predictor of depression score (β = 9.515; *p* < 0.001) and presence of depression (OR = 5.24; *p* < 0.001) (Tables 2 and 3).

**Table 1:** Sample characteristics

Variables	
Gender	
Male	105 (65.3)
Female	56 (34.7)
Age	50.3 ± 17.0
Economic class	
A	0
B	9 (5.6)
C	91 (56.5)
D	56 (34.8)
E	5 (3.1)
Marital status	
Married	118 (73.3)
Religion	
Catholic	128 (79.5)
Protestant or evangelical	27 (16.8)
No religion	6 (3.7)
End-stage renal disease etiology	
Hypertension	55 (34.2)
Glomerulonephritis	41 (25.2)
Diabetes	35 (21.7)
Obstructive uropathy	13 (8.0)
Polycystic kidney disease	6 (3.7)
Lupus	3 (1.9)
Undetermined	8 (5.0)
Months on dialysis	46.2 ± 51.5
Vascular access	
Fistula	130 (80.7)
Catheter	31 (19.3)
Comorbidity (Khan index)	
Low	82 (50.9)
Medium	59 (36.7)
High	20 (12.4)
Religious/spiritual scores (1–5)	
Positive religious/spiritual coping score	2.92 ± 0.55
Negative religious/spiritual coping score	1.78 ± 0.42
Depression score (0-60) (cut off for depression ≥18)	10.6 ± 9.4
Depression	
Yes	44 (27.3)
No	117 (72.7)
Quality of life scores (0-100)	
Physical function	45.4 ± 28.8
Role-physical	34.3 ± 38.2
Bodily pain	61.7 ± 26.9
General health	53.6 ± 21.8
Vitality	62.7 ± 20.8
Social functioning	69.3 ± 26.4
Role-emotional	70.4 ± 64.5
Mental health	75.2 ± 19.2
Laboratory findings	
Creatinine level (mg/dL)	8.4 ± 2.5
Hemoglobin level (g/dL)	9.3 ± 2.2
Albumin level (g/dL)	4.1 ± 0.4
Calcium-phosphorus product level (mg^2^/dL^2^)	44.6 ± 10.1
Kt/V index	1.7 ± 0.6

Data are reported as the means ± SD or numbers with percentages in parentheses

**Table 2:** Multivariate linear regression analysis of predictors of depression score

Predictors	β (Regression coefficient)	*P*
Gender (female)	3.341	0.020
Age	−0.050	0.282
Time on dialysis	0.011	0.415
Comorbidity index	3.255	0.004
Positive religious/spiritual coping score	−6.635	0.004
Negative religious/spiritual coping score	9.515	< 0.001

**Table 3:** Multivariate logistic regression analysis of predictors of depression

Predictors	OR	95% CI	*P*
Gender (female)	0.32	0.15−0.74	0.007
Age	0.99	0.96−1.02	0.525
Time on dialysis	0.99	0.98−1.01	0.856
Comorbidity index	1.60	0.84−3.06	0.148
Positive religious/spiritual coping score	0.13	0.02−0.91	0.039
Negative religious/spiritual coping score	5.24	2.18−12.60	<0.001

Regarding R/S coping methods and QOL, positive correlations were identified between positive R/S coping scores and both general health scores (*r* = 0.171; *p* = 0.030) and vitality scores (*r* = 0.183; *p* = 0.019), and inverse correlations were identified between negative R/S coping scores and both social functioning scores (*r* = −0.191; *p* = 0.015) and mental health scores (*r* = −0.214; *p* = 0.006) (Table 4). Positive R/S coping scores were identified as an independent predictor of both bodily pain (β = 14.401; *p* = 0.048) and vitality (β = 12.580; *p* = 0.022) scores, while negative R/S coping scores independently predicted scores in the social functioning domain (β = −21.158; *p* = 0.017) (Table 5).

**Table 4:** Pearson coefficients for the correlations between quality of life scores and positive and negative religious/spiritual (R/S) coping scores

Quality of life dimensions	Positive R/S coping score		Negative R/S coping score	
	R (correlation coefficient)	*P*	r (correlation coefficient)	*P*
Physical function	−0.092	0.245	−0.111	0.161
Role-physical	−0.106	0.178	−0.069	0.383
Bodily pain	0.142	0.072	−0.052	0.506
General health	0.171	0.030	−0.122	0.123
Vitality	0.183	0.019	−0.111	0.160
Social functioning	0.043	0.580	−0.191	0.015
Role-emotional	0.011	0.884	−0.125	0.113
Mental health	−0.002	0.978	−0.214	0.006

**Table 5:** Multivariate linear regression analyses of predictors of quality of life dimension scores

Quality of life dimensions	Predictors	β (Regression coefficient)	*P*
Physical function	Age	−0.627	<0.001
Role-physical	Age	−0.556	0.008
Bodily pain	Positive religious/spiritual coping score	14.401	0.048
General health	Comorbidity index	−9.168	0.001
Vitality	Gender (female)	8.365	0.001
	Positive religious/spiritual coping score	12.580	0.022
Social functioning	Negative religious/spiritual coping score	−21.158	0.017
Role-emotional	(no significant predictors)		
Mental health	Gender (female)	8.319	0.008
	Comorbidity index	−5.525	0.024

## Discussion

R/S coping methods have been well studied in medicine, and their connections with several health outcomes among chronic disease patients in general are well known [22–24]. Positive R/S coping methods may be associated with better perception of life, shortened length of hospitalization, decreased mortality and improved immune function [25–27]. There is even anatomical evidence suggesting that religion and spirituality are associated with a thicker brain cortex, which in turn may confer resilience to the development of depressive symptoms in individuals at high family risk for major depression [28]. Despite the wide body of scientific evidence, the religiosity and spirituality of patients are not usually addressed by health professionals in daily practice. For instance, a survey of Brazilian physicians found that only 4% received collegiate training regarding religion and spirituality in medicine [11]. In addition, the results of another study indicated that 74.1% of patients undergoing HD had not been asked about their religion by their physicians at the dialysis center [29]. Thus, studies about R/S coping methods are undoubtedly needed in the field of nephrology.

The instrument (RCOPE) used in this study to assess the included patients’ R/S coping methods encompasses religiosity from both the organizational perspective (when one believes in, follows or practices a religion through regular church or temple attendance) and from the perspective of spirituality experienced in a non-organizational manner, through regular prayer, reading books, watching religious programs on television, or considering sacredness or transcendence to explain the existential questions of life. In this sense, the two types of R/S coping methods (positive and negative) were strongly associated with the occurrence and intensity of depression and QOL, particularly patient vitality and social functioning.

### R/S coping and depression

In our study, R/S coping methods were associated with depression in many ways. First, positive R/S coping scores were lower, while negative R/S scores were higher among depressed patients. These differences were small, but differences of only decimals were validated as clinically significant during the process of development and validation of the RCOPE [14]. Second, there was an inverse correlation between positive R/S coping scores and depression scores, indicating that as the use of positive religious coping methods increase, depression scores tended to decrease. On the other hand, we identified a positive correlation between negative R/S coping scores and depression scores, suggesting that as the use of negative religious coping methods increases, depression scores tend to increase. Third, both positive and negative R/S coping methods were independent predictors of both CES-D scores and the presence of depression. Indeed, negative R/S coping scores were associated with a fivefold increase in the odds of depression in our sample. This finding, which indicated the presence of a correlation between religious aspects and depression, corroborates the results of another Brazilian study [30]. Among ESRD patients, depression is classified as compound depression, which indicates depression occurring in someone with another illness or medical condition. In these cases, resistance to treatment is more likely [5]. Thus, religiosity can be seen as a target for interventions. Multidisciplinary teams at dialysis centers can act, especially through social workers and psychologists, to support and stimulate various types of non-organizational religious activities that are known to promote the use of positive R/S coping methods, such as the reading of religious literature, praying or engagement in spiritual discussion groups. Organizational religious activities may be even more effective because church attendance strengthens patients’ social support systems through interactions with community members, faithful laypersons, and religious leaders. Previous studies have reported that that social support is a mediator of depressive symptoms in HD patients [31] and that religious affiliation is associated with lower rates of depression in some groups [32].

### R/S coping and quality of life

R/S coping methods were also associated with HD patients’ perception of their QOL. We identified positive correlations between positive R/S coping scores and scores related to general health and vitality, and inverse correlations between negative R/S coping scores and scores related to social functioning and mental health. Vitality and social functioning are worthy of further discussion because in addition to the bivariate correlations between scores, vitality and social functioning scores were predicted by positive R/S coping and negative R/S coping scores, respectively, in the multivariate analyses. One study found that R/S coping methods explained nearly 40% of the variance in QOL scores among Muslim patients undergoing HD [33]. The associations between R/S coping methods and both vitality and social functioning has been reported in different cultures. In Taiwan, a study also using the SF-36 found that patients undergoing HD presented higher social functioning scale scores if they had stronger spiritual beliefs when compared with those with no or weak spiritual beliefs (assessed by the Royal Free Interview for Spiritual and Religious Beliefs) [34]. In Canada, a study of HD patients found vitality and social functioning (as assessed using the SF-36) to be positively associated with existential well-being (as assessed using the Spiritual Well-Being Scale) [35]. In Iran, vitality and social functioning scores were lower among HD patients classified as having moderate spiritual well-being relative to those assessed as having high spiritual well-being according to the Spiritual Well-Being Scale [36]. In another study of Brazilian patients in which a brief version of the RCOPE was used, positive R/S coping scores were associated with better social functioning, as assessed using the WHOQOL-BREF, and negative R/S coping was associated with worse social functioning [30]. Based on these convergent results in these two dimensions of QOL, R/S coping can be a target for interventions aiming to improve vitality and social functioning of HD patients. In one interventional study, HD patients were invited to attend weekly meetings with a professional responsible for spiritual support, and after the intervention, a significant improvement in the participants’ QOL was identified [13]. In our opinion, similar results may be obtained if religious concerns are addressed by nephrologists, nurses, social workers and psychologists in dialysis centers.

### Limitations

The main limitation of our study is its cross-sectional design, which precluded the detection of the direction of causality between R/S coping and depression and QOL. We are aware that coping methods are dynamic and that the chronic disease itself, health status and psychological distress can cause changes in patient coping over time. For this reason, longitudinal studies are necessary, especially interventional studies that can show the effects of changes in R/S coping methods on outcomes. Another limitation is the particularities of our sample, which include the following: the participants were rarely diabetic, had low comorbidity indices, were of low economic class and were mostly Catholic. These factors preclude the generalizations of these data to more typical HD samples (older patients, diabetic patients with higher rates of comorbidity) and/or with other religious backgrounds.

### Implications

In summary, multidisciplinary teams at dialysis centers should interact with patients to minimize religious struggles and stimulate and/or support positive R/S coping methods in an effort to improve QOL and decrease depression among ESRD patients undergoing HD. The feasibility of these interactions strongly depends on the cultural background of the sample. This type of intervention would be difficult to implement in a sample consisting mostly of secular individuals.

## Conclusions

Our results provide further evidence suggest that religious and spiritual methods of coping may be associated with QOL and depression among HD patients. These findings have clinical implications for the care team, who can try to mitigate depressive symptoms and improve patients’ QOL by encouraging patients to utilize religious resources and incorporating psycho-spiritual interventions to minimize religious struggles (negative R/S coping) among HD patients.

## Abbreviations

CES-D: The Center for Epidemiologic Studies Depression Scales
ESRD: End-stage Renal Disease
HD: Hemodialysis
QOL: Quality of Life
R/S: Religious/Spiritual
RCOPE: Religious Coping Questionnaire
SF-36: Medical Outcomes Study 36-Item Short Form Health Survey

## References

[1] Cukor D, Cohen SD, Peterson RA, Kimmel PL (2007). Psychosocial aspects of chronic disease: ESRD as a paradigmatic illness. J Am Soc Nephrol.

[2] Mittal K, Ahern L, Flaster E, Maesaka JK, Fishbane S (2001). Self-assessed physical and mental function of haemodialysis patients. Nephrol Dial Transplant.

[3] Santos PR, Arcanjo FPN (2013). Social adaptability and substance abuse: predictors of depression among hemodialysis patients?. BMC Nephrol.

[4] Gabbay E, Meyer KB, Griffith JL, Richardson MM, Miskulin DC (2010). Temporal trends in health-related quality of life among hemodialysis patients in the United States. Clin J Am Soc Nephrol.

[5] Iosifescu DV, Nierenberg AA, Alpert JE, Smith M, Bitran S, Dording C (2003). The impact of medical comorbidity on acute treatment in major depressive disorder. Am J Psychiatry.

[6] Santos PR (2010). Correlation between coping style and quality of life among hemodialysis patients from a low-income area in Brazil. Hemodial Int.

[7] Tsay SL, Lee YC, Lee YC (2005). Effects of an adaptation training programme for patients with end-stage renal disease. J Adv Nurs.

[8] Santos PR, Capote JRF, Cavalcanti JU, Vieira CB, Rocha AR, Apolônio NA (2013). Sexual dysfunction predicts depression among women on hemodialysis. Int Urol Nephrol.

[9] Peterman AH, Fitchett G, Brady MJ, Hernandez L, Cella D (2002). Measuring spiritual well-being in people cancer: the functional assessment of chronic illness therapy-spiritual well-being scale. Ann Behav Med.

[10] Smith TB, McCullough ME, Poll J (2003). Religiousness and depression: evidence for a main effect and the moderating influence of stressful life events. Psychol Bull.

[11] Lucchetti G, Ramakrishnan P, Karimah A, Oliveira GR, Dias A, Rane A (2016). Spirituality, religiosity, and health: a comparison of physicians’ attitudes in Brazil, India, and Indonesia. Int J Behav Med.

[12] Trevino KM, McConnell TR (2014). Religiosity and religious coping in patients with cardiovascular disease: change over time and associations with illness adjustment. J Relig Health.

[13] Weisbord SD, Carmody SS, Bruns FJ, Rotondi AJ, Cohen LM, Zeidel ML (2003). Symptom burden, quality of life, advance care planning and the potential value of palliative care in severely ill haemodialysis patients. Nephrol Dial Transplant.

[14] Pargament KI, Koenig HG, Perez LM (2000). The many methods of religious coping: development and initial validation of the RCOPE. J Clin Psychol.

[15] Panzini RG, Bandeira DR (2005). Escala de coping religioso-espiritual (Escala CRE): elaboração e validação de construto. Psicol Estud.

[16] Radloff LS (1977). The CES-D scale: a self-report depression scale for research in the general population. Appl Psychol Meas.

[17] Hedayati SS, Bosworth HB, Kuchibhatla M, Kimmel PL, Szczech LA (2006). The predictive value of self-report scales compared with physician diagnosis of depression in hemodialysis patients. Kidney Int.

[18] Cicconelli RM, Ferraz MB, Santos W, Meinão I, Quaresma MR (1999). Brazilian-Portuguese version of the SF-36: a reliable and valid quality of life outcome measure. Rev Bras Reumatol.

[19] Brazilian Association of Research Institutes. Classification of socioeconomic status. http://www.abep.org/criterio-brasil. Accessed 26 Mar 2015.

[20] Khan IH, Campbell MK, Cantarovich D, Catto GR, Delcroix C, Edward N (1996). Survival on renal replacement therapy in Europe: is there a “centre effect”?. Nephrol Dial Transplant.

[21] Daugirdas JT (1993). Second generation logarithmic estimates of single-pool variable volume Kt-V: an analysis error. J Am Soc Nephrol.

[22] Bonelli RM, Koenig HG (2013). Mental disorders, religion and spirituality 1990 to 2010: a systematic evidence-based review. J Relig Health.

[23] Kirby SE, Coleman PG, Daley D (2004). Spirituality and well-being in frail and nonfrail older adults. J Gerontol B Psychol Sci Soc Sci.

[24] Narayanasamy A (2002). Spiritual coping mechanisms in chronically ill patients. Br J Nurs.

[25] Panzini RG, Rocha NS, Bandeira DR, Fleck MPA (2007). Qualidade de vida e espiritualidade. Rev Psiq Clin.

[26] Koenig HG, Larson DB, Hays JC, McCullough ME, George LK, Branch PS (1990). Religion and survival of 1010 male veterans hospitalized with medical illness. J Relig Health.

[27] Koenig HG, Cohen HJ, George LK, Hays JC, Larson DB, Blazer DG (1997). Attendance at religious services, interleukin-6, and other biological indicators of immune function in older adults. Int J Psychiatry Med.

[28] Miller L, Bansal R, Wickramaratne P, Hao X, Tenke CE, Weissman MM (2014). Neuroanatomical correlates of religiosity and spirituality: a study in adults at high and low familial risk for depression. JAMA Psychiatry.

[29] Lucchetti G, Almeida LGC, Granero AL (2010). Spirituality for dialysis patients: should the nephrologist address?. J Bras Nefrol.

[30] Ramirez SP, Macêdo DS, Sale PMG, Figueiredo SM, Daher EF, Araújo SM (2012). The relationship between religious coping, psychological distress and quality of life in hemodialysis patients. J Psychosom Res.

[31] Khalil AA, Abed MA (2014). Perceived social support is a partial mediator of the relationship between depressive symptoms and quality of life in patients receiving hemodialysis. Arch Psychiatr Nurs.

[32] Bonelli R, Dew RE, Koenig HG, Rosmarin DH, Vasegh S. Religious and spiritual factors in depression: review and integration of the research. Depress Res Treat. 2012;962860 10.1155/2012/962860 PMC3426191 22928096

[33] Saffari M, Pakpour AH, Naderi MK, Koenig HG, Baldacchino DR, Piper CN (2013). Spiritual coping, religiosity and quality of life: a study on Muslim patients undergoing haemodialysis. Nephrology.

[34] Kao T, Chen P, Hsieh C, Chiang HW, Tsang LY, Yang IF (2009). Correlations between spiritual beliefs and health-related quality of life of chronic hemodialysis patients in Taiwan. Artif Organs.

[35] Davison SN, Jhangri GS (2010). Existential and religious dimensions of spirituality and their relationship with health-related quality of life in chronic kidney disease. Clin J Am Soc Nephrol.

[36] Kharame ZT, Zamanian H, Foroozanfar S, Afsahi S (2014). Religious wellbeing as a predictor for quality of life in Iranian hemodialysis patients. Glob J Health Sci.

